# Abortive Abscesses

**Published:** 1889-12

**Authors:** 


### Aborting Abscesses.

Apply a yeast poultice to the affected parts, upon which equal parts of borate of soda, boric acid, salicylic acid and powdered tannin should be dusted.

A moderate dose of calomel should be given internally. This treatment is usually sufficient to abort an abscess if it is resorted to when the local symptoms first make their appearance.

Frictions with the following ointment will also be found valuable:

| | | |
|---|---|---|
| ℞ | Salicylate of bismuth | 2½ drachms. |
| | Lanoline | 7½ drachms. |

—*Le Bulletin Med.*, September 29, 1889.

